# Toward a universal metasurface for optical imaging, communication, and computation

**DOI:** 10.1515/nanoph-2022-0155

**Published:** 2022-08-22

**Authors:** Prachi Thureja, Ruzan Sokhoyan, Claudio U. Hail, Jared Sisler, Morgan Foley, Meir Y. Grajower, Harry A. Atwater

**Affiliations:** Thomas J. Watson Laboratory of Applied Physics, California Institute of Technology, Pasadena, CA 91125, USA

**Keywords:** active metasurface, space-time modulation, universal element, wavefront shaping

## Abstract

In recent years, active metasurfaces have emerged as a reconfigurable nanophotonic platform for the manipulation of light. Here, application of an external stimulus to resonant subwavelength scatterers enables dynamic control over the wavefront of reflected or transmitted light. In principle, active metasurfaces are capable of controlling key characteristic properties of an electromagnetic wave, such as its amplitude, phase, polarization, spectrum, and momentum. A ‘universal’ active metasurface should be able to provide independent and continuous control over all characteristic properties of light for deterministic wavefront shaping. In this article, we discuss strategies for the realization of this goal. Specifically, we describe approaches for high performance active metasurfaces, examine pathways for achieving two-dimensional control architectures, and discuss operating configurations for optical imaging, communication, and computation applications based on a universal active metasurface.

## 1 Introduction

The first reported optical lens, dating to the Ancient Egyptian era (2620 BC), employed convex polished rock crystals to form the eyes of ornate statues [1]. The unique function of these lenses enabled the eyes to appear to ‘follow’ the viewer as they moved around the statue [2]. Ever since, scientists have strived to understand and control the properties of light, leading to a variety of advanced technologies that are now fundamental to society. Prominent examples include the invention of the telescope for ‘seeing things far away as if they were nearby’ [3] and the development of the first photographic camera to capture the visual information in a scene. In these constructs, optical wavefronts are controlled using bulky optical elements which have a thickness of few millimeters. Today, with the large advances in micro- and nanofabrication in recent decades, researchers are developing next-generation flat optical components that mold the wavefront of light at a subwavelength scale, giving rise to versatile and unprecedented control over light.

Metasurfaces are composed of two-dimensional (2D) arrays of subwavelength resonant scatterers, which locally and abruptly change the characteristic properties of the impinging light, i.e., its amplitude, phase, polarization, spectrum, and momentum. This also results in control of the far-field properties of light, giving rise to anomalous reflection [4, 5], polarization control [6], or beam focusing [7–9], which result from constructive interference of the waves that are scattered collectively by the entire array. Initial efforts at wavefront control employed passive metasurfaces, in which individual scatterers were geometrically designed to create the desired local change in electric field (e.g., a phase offset) at the working wavelength. Using this approach, researchers have demonstrated multifunctional passive metasurfaces [10, 11] that can perform several tasks simultaneously, otherwise only achievable *via* combination of multiple bulk optical components. A fundamental limitation which arises due to the static nature of passive metasurfaces is that they do not allow for any post-fabrication tunability. Consequently, the use of passive metasurfaces is restricted to specific, pre-defined functions. Many modern applications in the realms of optical imaging, communication, and computation, however, require dynamically tunable building blocks that allow for on-demand wavefront shaping. Examples of this include eye tracking for augmented reality headsets or remote sensing in autonomous vehicles.

Recently, considerable effort has been devoted to the realization of reconfigurable, active metasurfaces [12–15]. In this case, the metasurface consists of an array of geometrically periodic, often identical unit cells. Dynamic control over the optical response of the metasurface is then obtained upon application of an external stimulus. This external stimulus can alter the resonant properties of the subwavelength scatterers *via* actively inducing a refractive index change in an active material layer integrated into the metasurface. Alternatively, the application of the external stimulus can deform the metasurface unit cell dimensions or the relative position of individual scatterers, resulting in dynamic control of the wavefront of light reflected or transmitted from the array. While active metasurfaces have been designed at operating wavelengths over a wide range of the electromagnetic spectrum, the following discussion aims to give a perspective on metasurfaces operating at visible and near-infrared (NIR) wavelengths.

To date, active tuning of metasurfaces has leveraged mechanical deformation of nanophotonic structures [16, 17], field-effect tuning [18, 19], electro-optic [20], thermo-optic [21], electrochemical [22] and chemical effects [23], structural changes in phase change materials [24, 25] and liquid crystals [26], as well as all-optical modulation schemes [27]. Moreover, multifunctional active metasurfaces, which can switch between multiple continuously tunable functions, have recently been demonstrated experimentally [28]. The basis for such devices lies in implementing an active metasurface with individually addressable unit cells. Using this concept, Kafaie Shirmanesh et al. [28] achieved diverse optical functions, such as dynamic beam steering and varifocal lensing, by changing the configuration of voltages applied onto a single field-effect tunable metasurface. By using specifically tailored metasurface designs and varying the external stimulus applied onto each metasurface unit cell, researchers have further managed to dynamically generate desired changes in the amplitude, phase, and polarization of the scattered electric field.

**Figure 1:**
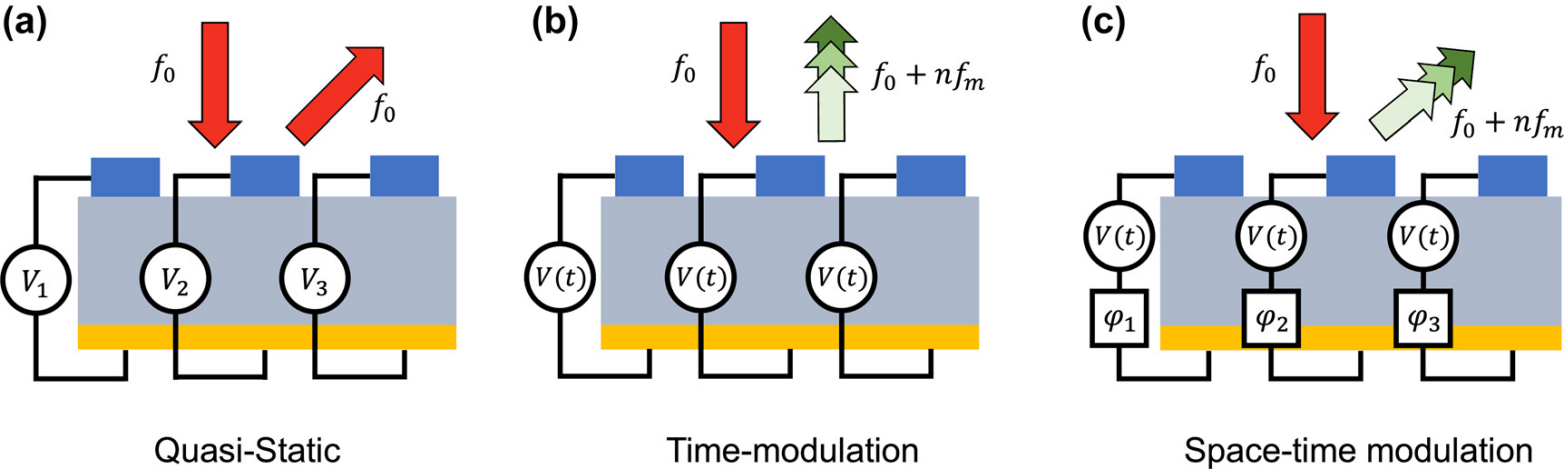
Incident light of frequency *f*_0_ is reflected from an electrically programmable metasurface. (a) In the quasi-static operation regime, the timescale of reconfiguration is smaller than the linewidth of the incident laser, such that the frequency of the reflected light remains unchanged. (b) In the time-modulated regime, metasurface elements are collectively driven at a high frequency, *f*_*m*_, allowing the generation of higher order harmonics in the reflected light with frequencies *f*_0_ + *nf*_*m*_. (c) In a space-time modulated metasurface, a phase offset *φ*_*i*_ is added between the time-modulated signals of individual metasurface elements using external phase shifters. This enables shaping of frequency-modulated reflected waves.

The timescale of active reconfiguration of a metasurface is an important characteristic determining its mode of operation. If the temporal rate of reconfiguration is significantly smaller than the optical frequency, the metasurface can be viewed as operating in the *quasi-static* regime (Figure 1a). Another regime, which we term the *time-modulated* regime, is reached once the metasurface elements are actuated with frequencies larger than the incident laser linewidth, which is less than 1 MHz for state-of-the-art lasers at optical wavelengths. In this case, the metasurfaces give rise to additional frequency harmonics that appear as sidebands, displaced in frequency relative to the incident laser frequency, as shown in Figure 1b. Individual frequency harmonics can further be controlled independently using space-time modulated metasurfaces (Figure 1c). Here, an additional phase offset is introduced between the high-frequency driving waveform of each metasurface element using external non-resonant phase shifters [29, 30]. This concept is of particular interest to optical communications applications. High-frequency time modulation gives the ability to increase the number of communication channels carrying information, similar to wavelength division multiplexing (WDM) in optical fiber communications [31].

**Figure 2:**
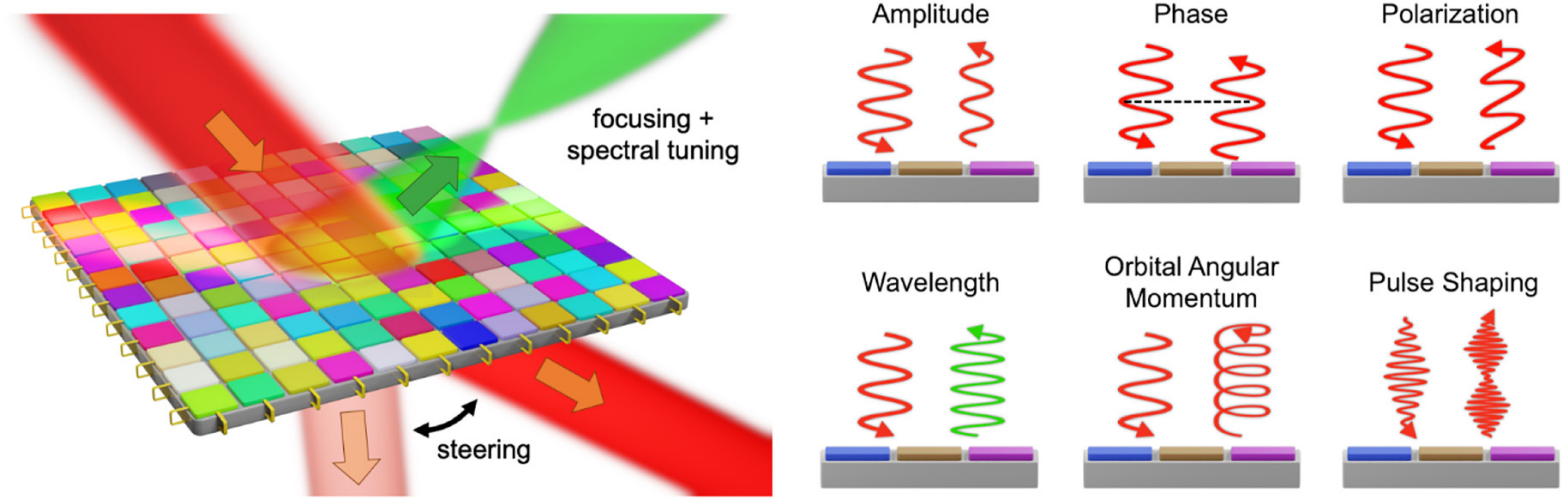
Schematic of a universal active metasurface enabling dynamic wavefront shaping in reflection and transmission. This metasurface should allow for dynamic and independent control of the amplitude, phase, polarization, wavelength, the orbital angular momentum, and pulse shape of an electromagnetic wave.

The ability to dynamically control the optical response in the quasi-static and time-modulated regimes opens a multidimensional design space that can be fully harnessed by developing appropriate nanophotonic structures for arbitrary manipulation of light. In this regard, the fundamental question that arises is: What does it take to realize a *universal* optical element (Figure 2) which enables dynamic, independent, and comprehensive control over all constitutive properties of light in both reflection and transmission? State-of-the-art wavefront shaping metasurfaces generally encompass active and continuous control over the amplitude and phase of light scattered from each nanostructured element. A universal active metasurface, in comparison, should additionally provide complete control over the polarization, spectrum and momentum, the orbital angular momentum as well as the shape of optical pulses. Such a universal active metasurface would have the potential to serve as a programmable transfer element that can encode arbitrary functions and perform a variety of complex tasks using a single dynamically tunable component. As such, it could be integrated into a wide range of applications, including free-space communications, analog computing, and holographic displays to name a few.

Optical phased arrays consist of an array of waveguides coupled to separate emitters [32]. As such, they are different from metasurfaces in that they form emitting apertures rather than nanophotonic components for far-field to far-field transformations. Real-time dynamic wavefront control has been achieved through phase shifters connected to each emitter. However, the sparse arrangement of waveguides for reduced crosstalk generally limits the attainable field-of-view. This limitation can be overcome using an aperiodic arrangement of emitters [33]. Nevertheless, for two-dimensional control, optical phased arrays often rely on a combination of phase control along one axis and wavelength tuning along the other axis [34, 35]. As a consequence, optical phased arrays have limited usage in advanced applications beyond beam steering, such as active focusing, beam shaping, polarization conversion and active orbital angular momentum control amongst others. In addition, we note that metasurfaces potentially have higher optical power handling capabilities compared to optical phased arrays [36].

In this article, we focus on the pathways toward achieving a universal active metasurface for independent and comprehensive, real-time control over all properties of light*,* including its amplitude, phase, polarization, momentum, spectrum, orbital angular momentum, and pulse shape. We identify key challenges and highlight the respective recent developments in three areas that will drive the realization and unlock the potential of a universal active metasurface: *metasurface design*, *control architecture*, and *advanced operation modes*. We outline the target performance characteristics with regards to a high-performance active metasurface design and discuss potential pathways for their realization. We further examine challenges toward realizing a two-dimensional control architecture and discuss strategies to overcome currently existing limitations. We then explore uncharted operation modes that could be attained with a universal metasurface, providing inspiration for future applications in the realms of optical imaging, communication, and computation. Finally, we give a perspective and point toward key technological problems that will require further research from the nanophotonics community to bring us closer to the realization of a truly universal active metasurface.

## 2 Progress & challenges

### 2.1 Metasurface design

The design of a universal active metasurface forms a convoluted problem involving several parameters. Passive metasurfaces generally rely on shape- and orientation-dependent phase retardation introduced by resonant scatterers to create desired changes in the scattered electric field. In comparison, the performance of an active structure is determined by its geometrical parameters as well as the choice of the active material and/or the corresponding modulation scheme. In the following, we review active metasurface design strategies that hold the potential to realize a universal active metasurface. The desired objectives can be outlined as follows: First, a large dynamic range of tuning is desired for deterministic wavefront shaping. Here, the objective is to obtain complete and independent control over the characteristic properties of light. This includes a 0-to-2*π* phase shift upon actuation (while maintaining constant amplitude) and 100% intensity modulation efficiency (with constant phase). We define the latter as *η* = 1 − *I*_min_/*I*_max_, where *I*_min_ and *I*_max_ are the minimal and maximal electric field intensity at a given wavelength, respectively. In addition, low loss structures are required to ensure high output efficiencies. To access the time-modulated operation regime, active metasurfaces further need to support large modulation frequencies. Additional performance metrics include broadband operation, allowing for a tunable excitation wavelength and achromaticity. While these criteria do not form an exhaustive list of requirements for a high-performance universal metasurface, they determine critical design choices for the architecture and materials employed in a metasurface unit cell as well as its modulation mechanism.

**Figure 3:**
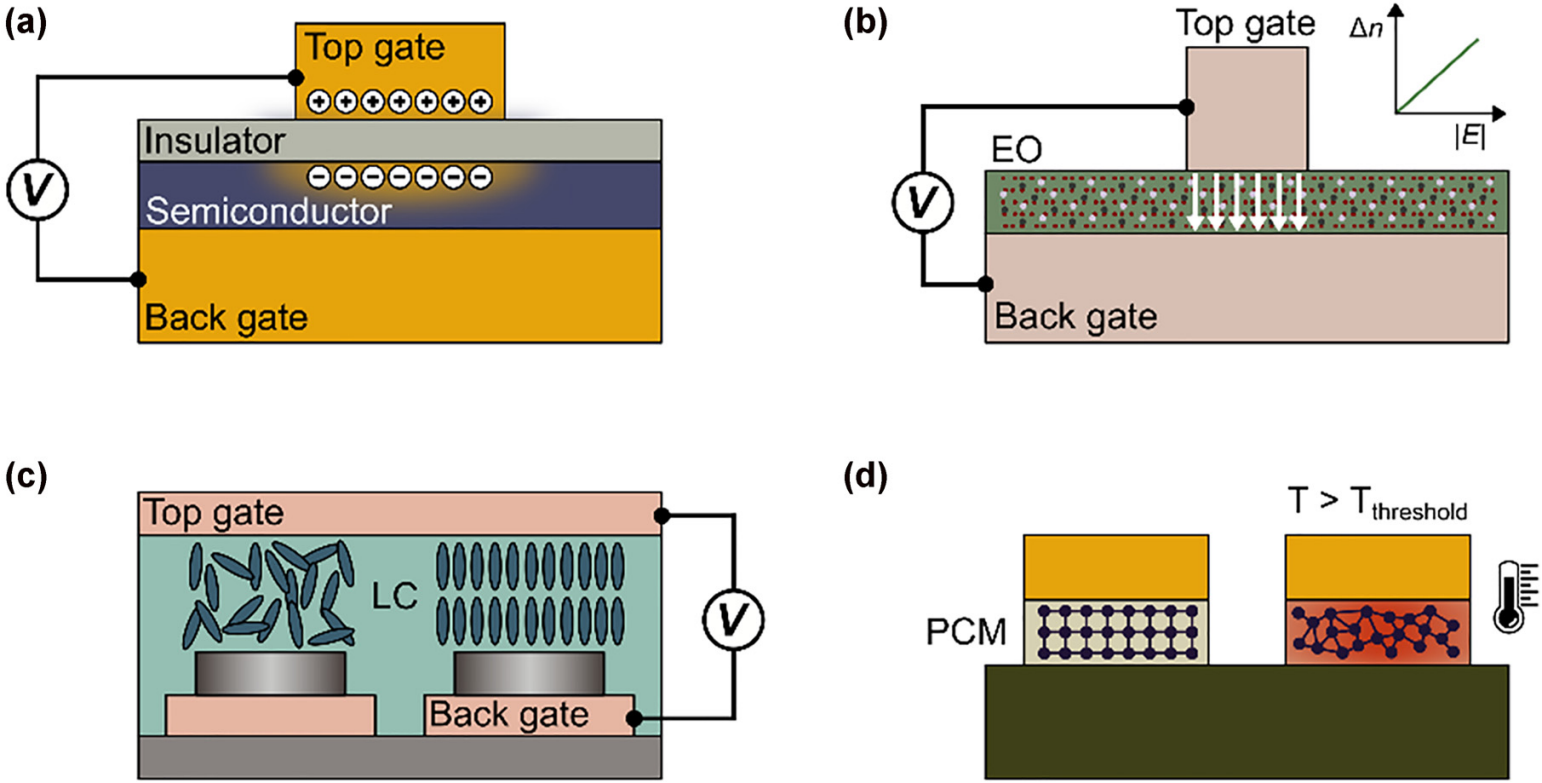
(a) Field-effect tunable metasurface. When the active semiconductor is integrated into a metal (or semiconductor)-oxide-semiconductor layer, a charge accumulation layer is formed upon application of a gate voltage. (b) Electro-optically tunable metasurface relying on Pockels effect. An electric field *E* (white arrows) applied across an electro-optic (EO) thin film of a non-centrosymmetric crystal leads to a linear change in refractive index Δ*n*. (c) Liquid crystal-based metasurface. The liquid crystal molecules (LC) reorient in an external electric field. (d) Active metasurface based on phase change materials (PCM). The active material undergoes a phase transition from crystalline to amorphous when heated above a threshold temperature *T*_threshold_.

Field-effect tuning based on carrier index modulation has attracted significant attention over the past decade, as it has been used to experimentally demonstrate a large dynamic range of tuning of amplitude, phase, and polarization. Individual studies have reported up to 96% reflectance modulation efficiency [37], >1.5*π* phase modulation [28, 38], [39], [40], as well as a linear to circular and cross-polarization conversion [41]. Field effect tuning relies on the charge carrier dependent optical properties in semiconductors, often transparent conductive oxides (TCOs), or in graphene. In semiconductor-based tuning, a metal (or semiconductor)-insulator-semiconductor heterostructure [18, 42] is integrated into a resonant unit cell. Upon gating, a local charge carrier accumulation or depletion zone is created at the insulator-semiconductor interface (Figure 3a), causing a change in the complex dielectric permittivity, *ε* [43, 44]. Additionally tuning the dielectric permittivity into the epsilon-near-zero (ENZ) regime leads to an extreme localization of the electric field in this zone, which perturbs the optical resonant mode of the unit cell [45]. This ability to transition the active material through the ENZ region, and hence dynamically modify the scattering of a resonant unit cell has led to the development of electronically programmable active metasurfaces with independently addressable metasurface elements [28, 46]. Moreover, modulation frequencies of up to 10 MHz have been demonstrated using TCO-based metasurfaces [18], with the potential of accessing GHz frequencies with optimized design of device electrical interconnects and driver circuits.

The operating wavelength of TCO metasurfaces is chosen based on the bulk carrier density of the active semiconductor during fabrication. Indium tin oxide (ITO) is a commonly used active material for operation in the near-infrared. A recent study, however, showed that over 70% of the incident light is absorbed in the ITO layer at its ENZ transition and only 2.7% of the light is reflected [36]. Cadmium oxide (CdO) was proposed as an alternative transparent conducting oxide with enhanced optical properties, leading to reflectance values above 22% [36]. For operation in the mid-infrared (3–10 μm), several materials including graphene, and doped semiconductors such as indium arsenide (InAs) [47] have been proposed. Phononic materials, such as silicon carbide (SiC) [48], indium phosphide (InP) [49] and gallium arsenide (GaAs) [50], may provide a promising pathway to realize field-effect tunable metasurfaces operating beyond 10 μm [51]. This class of materials supports a phonon-polariton mode that undergoes an ENZ transition in the far-infrared while maintaining a low extinction coefficient. For operation in the visible, transition metal nitrides stand out as an emerging class of materials that undergo an ENZ transition between 400 and 800 nm [51, 52].

Atomically thin polar van der Waals (vdW) materials and transition metal dichalcogenides (TMDCs) offer an alternative pathway to extend the operation wavelength of active metasurfaces to the visible spectrum. These materials support strong excitonic resonances at wavelengths determined by the dielectric properties of the TMDC material. By integrating the 2D semiconductor material into a capacitive structure and biasing the gate electrode, charges can be injected into the active layer. Due to the exciton-charge interaction, a modification of the excitonic resonance is observed [53–55]. As a result, a large change in refractive index can be achieved [56, 57]. The index changes at excitonic resonances manifest themselves in phase and amplitude modulation even without explicit optical resonators. An integration into planar heterostructures forming resonant cavities further enhances the observed dynamic range of tuning [58–60]. Recently, black phosphorus (BP) was proposed as an excitonic material suitable for active metasurfaces. The intrinsic anisotropy along the crystal axes [61] of BP allows for dynamic polarization control [62]. Currently, patterning of 2D materials and integration into metasurface building blocks is being pursued to further enhance light–matter interactions and enable advanced optical functions [63]. Recent experimental demonstrations of this include a tunable zone plate lens allowing for a modulation of the focal intensity [64] as well as a nanostructured array for dynamic control of the scattering pattern, supporting modulation frequencies of up to 625 MHz [65].

Despite the progress on the dynamic modulation achieved using field-effect tunable metasurfaces, there are several limitations that need to be overcome for use in applications. One of the challenges with metasurfaces based on thin films of excitonic materials is the scalability of devices to large areas (greater than 500 μm^2^). The highest quality 2D semiconductor films are obtained through mechanical exfoliation. This approach becomes especially challenging when multiple layers need to be stacked on top of each other. Recent advances with robotically assisted thin film transfer [66, 67] may provide a potential pathway toward realizing scalable active heterostructures. Nevertheless, current TMDC-based active structures require cryogenic temperatures to realize their full dynamic range of tuning [65]. Additionally, while the operating wavelength of TCO-based metasurfaces can be changed to some degree *via* an appropriate choice of material doping, the operating wavelength range for TMDC-based structures is intrinsically fixed by the excitonic transition of the material. Field-effect tunable metasurfaces further require a high electric field confinement and mode overlap of their active media with resonant nanocavity structures to obtain strong light–matter interactions. This can be achieved *via* plasmonic resonators and cavities, which, however, contribute to high absorption and thus lower device efficiencies. Alternatively, a double distributed Bragg reflector cavity was proposed to enhance light–matter interactions in a BP-based planar heterostructure [68]. Using this design, a phase modulation of 300° was predicted along with a minimum reflectance above 50% at an operating wavelength of 2.9 μm, where BP exhibits lower losses. Further research is required to explore a potential extension of this platform to an active metasurface with independently addressable unit cells.

Electro-optic tuning of metasurfaces offers a promising approach to low-loss metasurfaces. The Pockels effect relies on a linear variation of refractive index in response to an applied electric field (Figure 3b) [69]. This is a broadband phenomenon which appears in non-centrosymmetric crystals, such as lithium niobate (LN) [70], barium titanate (BTO) [71], and aluminum nitride (AlN) [72] amongst others. Electro-optically tunable materials further support ultrafast modulation frequencies (>100 GHz in electro-optic modulators [73]). Generally, polycrystalline or amorphous thin film materials exhibit smaller electro-optic coefficients compared to single crystal materials and thus result in a smaller refractive index variation. The reduction in electro-optic coefficient strongly depends on the growth method. The electro-optic coefficients of BTO and LN grown using metalorganic deposition techniques, for example, are typically up to 3–4 times smaller compared to their bulk counterparts [71, 74]. However, commercially available high-quality LN thin films have recently propelled the integration of electro-optic materials into active metasurfaces [75, 76]. Another significant advance entailed a recent demonstration of epitaxially grown thin-film BTO with an approximately 30x larger electro-optic coefficient compared to LN [77]. Reports of active metasurfaces integrating BTO as a tunable layer, however, have thus far been limited to lower electro-optic coefficients [78]. Organic electro-optic (OEO) chromophores, in comparison, provide a promising pathway for low-cost, high-throughput integration using methods such as spin-coating, micro-dispensing or ink-jet printing [79–81]. They have additionally enabled a strong modulation of the transmitted intensity at frequencies up to 5 GHz [82]. However, we note that high electric field intensities may cause photo degradation of the chromophores due to the presence of oxygen in the active material [83]. Besides the Pockels effect, the quantum confined Stark effect offers an alternative route to obtain a fast, electro-optically tunable response. In this case, a change in refractive index is induced upon an applied electric field, which causes a shift in the interband transition energy [84–86]. Based on this principle, Wu et al. [87] recently proposed an all-dielectric active metasurface based on multiple quantum wells (MQWs). In this work, active beam switching was realized with a phase shift of 70° and a simultaneous reflectance modulation efficiency of *η* ∼ 73%.

Table 1 provides a summary of the electro-optic refractive index change obtained in various nanophotonic devices using the active dielectric materials highlighted above. Due to a modest index change, the active electro-optic layer generally needs to be coupled to a high-quality (*Q*) resonant mode to obtain a strong modulation of the scattered wavefront using an active metasurfaces. To this end, several metasurface designs relying on guided mode resonances have been realized [87, 88]. However, in this case, the extended mode along one dimension complicates the design of compact resonant elements for two-dimensional metasurface arrays with independently addressable unit cells. Active metasurfaces governed by optical bound states in the continuum (BICs) or quasi-BICs [89–91] may offer another promising pathway to incorporate electro-optic materials into high-*Q* resonant structures [92] and thus represent one approach to the design of non-local active metasurfaces [93]. Non-local metasurfaces rely on a collective mode generated by an array or subset of metasurface elements. Similarly, Weiss et al. [76] proposed a design strategy relying on the mode overlap of a Fabry–Pérot resonance, a localized surface plasmon resonance, and a surface lattice resonance. Notably, the surface lattice resonance only appears when the array periodicity coincides with the localized surface plasmon resonance of the metasurface elements. Therefore, further investigations are required to analyze whether this modulation scheme can be extended to gain active control on an individual unit cell level.

**Table 1:** Refractive index change Δ*n* obtained through experimentally reported values of the electro-optic coefficient and the applied field strength for a variety of thin-film electro-optic materials and multiple quantum wells integrated into nanophotonic devices. The wavelength coverage corresponds to the transparency window of different electro-optic materials.

Active electro-optic material	Electro-optic	Applied field	change Δ*n*	Wavelength
	coefficient	strength		coverage
	[pm/V]	[MV/cm]		
Aluminum nitride (AlN)	*r*_13_ = 0.67 [94]	1.8 [72]	0.0006	300 nm–8 μm [95]
Lithium niobate (LN)	*r*_33_ = 30.8 [96]	0.15 [97]	0.0022	420 nm–5.2 μm [98]
Organic electro-optic (OEO)	*r*_33_ = 359 [79, 99]	0.05 [100]	0.005	Above 1.2 μm^c^ [101]
chromophore JRD1				
Barium titanate (BTO)	*r*_42_ = 923 [77]	0.002 [77]	(0.0634)^a^	375 nm [102]–7 μm [103]
Multiple quantum wells (MQW)	N/A	0.1 [87]	∼0.002^b^ [104, 105]	915–960 nm [87], 1.41–1.44 μm [104],
				1.53–1.57 μm [105]

^a^The observed effective refractive index change in the BTO film will differ from this value due to the specifics of the orientation of different ferroelectric domains. ^b^We report the index change values in the spectral region where the extinction coefficient *k* is relatively small. ^c^To identify the upper bound of the operating wavelength, additional studies are needed.

Liquid crystals are well understood materials that have been widely deployed in display applications, and thus offer another active medium for realizing active metasurfaces with individually addressable unit cells. Here, a tunable optical response is obtained due to the optical birefringence of the liquid crystal molecules, which reorient upon application of a thermal [26, 106] or electrical stimulus (Figure 3c) [107, 108]. The corresponding large refractive index change ranges from Δ*n* = 0.15–0.4 across the visible and infrared spectrum (*λ* ∼ 460 nm–80 μm) [108, 109]. A commonly used active metasurface design approach relies on Huygens’ scatterers which are based on the spectral overlap of electric and magnetic dipoles. By actuating individual unit cells using an electrical bias, the liquid crystal orientation can be locally modified, as shown by Li et al. [108] in a transmissive liquid crystal-based metasurface. Alternatively, an active reflective liquid-crystal structure was recently realized based on Fabry–Pérot nanocavities that support multiple resonances across the visible spectrum [110]. Here, a continuously tunable phase shift across 2*π* is obtained while maintaining a high reflectance above 40%. It is worth noting that as the metasurface pitch is reduced to values below 1 μm, crosstalk between unit cells arises due to the elastic motion of liquid crystals [108, 111]. Furthermore, due to the response time of the molecules, the modulation frequencies are limited to several kHz, with currently fastest rates of 40 kHz reported in commercial light detection and ranging (LiDAR) devices [112].

Phase change or phase transition materials, by contrast, produce structural changes in the active layer upon Joule heating (Figure 3d), and take advantage of the large difference in complex refractive index achievable in different phases of the same material. Phase change materials, such as germanium-antimony-telluride (GST) and its derivatives [24, 113], undergo a non-volatile transition from an amorphous state to a crystalline state. Phase transition materials, on the other hand, rely on a volatile insulator-to-metal transition in materials such as vanadium dioxide (VO_2_) [25, 114]. Both material categories have been integrated into active structures exhibiting multi-level phase tuning. Experimental realizations of active metasurfaces using an electrical stimulus, however, have thus far been limited to modulation frequencies of 3 kHz [114]. Optical control has enabled significantly faster modulation rates, with amorphization in GST induced upon 50 fs long laser pulses [115]. (Notably, the recrystallization required repetitive pulsing of the fs laser for 1 s at 960 Hz.) Furthermore, several metasurface designs realizing tunable functions, such as active beam switching [114, 116] and bifocal lensing [116], have been demonstrated using phase change and phase transition materials. Nevertheless, the implementation of a metasurface with independently addressable elements requires further investigations on thermal heat management. In a preliminary study, Kim et al. [117] theoretically showed that thermal crosstalk between adjacent elements in a VO_2_-based metasurface could potentially be mitigated by incorporating heat conduction layers into the design.

High-performance active metasurfaces comprise structures that allow independent and comprehensive control over all properties of light. A full 2*π* phase coverage with close to unity amplitude has previously been obtained through strategies such as coupling of a resonator to a back reflector [88]. Additional independent control over the amplitude was attained using dual-gated structures [38], which can cover the entire complex amplitude space based on distinct voltage configurations [39, 40]. Notably, however, the design of compact and efficient active metasurfaces becomes increasingly challenging with the addition of each degree of freedom. At each step, the design problem constitutes a thorough co-optimization of the active material, the geometrical parameters of the metasurface element, as well as the external control variable. Furthermore, it becomes crucial to weigh trade-offs between different objectives for a desired application. As such, the task of finding an optimally functioning universal active metasurface is ideally formulated as an inverse design problem.

While inverse design has been widely explored for the geometrical optimization of unit cells in passive metasurfaces [118–120], active metasurfaces present a unique challenge in which the performance of a device must be optimized at multiple states simultaneously [121]. This concept of multi-state optimization has been theoretically demonstrated to optimize the function of active metasurfaces based on phase change materials [121, 122] or liquid crystals [123], which can switch between two states. In these studies, a shape or topology optimization was conducted on a single unit cell level using multi-objective optimization algorithms. For the design of a continuously tunable metasurface, however, the number of operation states of a metasurface dramatically increases. This challenge is further amplified with the independent addressability of metasurface elements. Several groups have thus employed a so-called array-level inverse design [124, 125]. Here, the value of the external bias is optimized at each element to overcome the limitations posed by a forward-designed active unit cell, which exhibits co-varying phase and amplitude and smaller than 2*π* phase shift upon actuation. We would like to note here that a common challenge in obtaining high-performance active metasurfaces is the degraded array performance despite working with structures that exhibit a large phase modulation and high reflectance. While some of these discrepancies can be attributed to fabrication imperfections, an important aspect that needs to be addressed is mutual coupling between neighboring metasurface elements [126].

Table 2 provides an overview of the performance of different modulation mechanisms in terms of the initially defined objectives. Field-effect tunable metasurfaces relying on carrier modulation and liquid crystal-based active structures stand out in terms of the achievable intensity modulation efficiency and the phase modulation. While field-effect tunable metasurfaces outperform in terms of the modulation frequency, liquid crystal-based architectures enable higher efficiency designs in both reflection and transmission. However, a drawback of current liquid-crystal based structures is the lack of subwavelength control in the visible due to mutual crosstalk. Meanwhile, metasurfaces relying on electro-optic tuning seem promising in terms of their efficiency and the accessible modulation frequencies. The operation wavelength can additionally be chosen from a broadband regime. However, the small index changes in the active material pose additional challenges to the realization of high-*Q* resonant metasurface unit cells at small dimensions needed for full two-dimensional control. Metasurfaces based on 2D materials or phase change/phase transition materials may provide an effective alternative to satisfy the desired objectives, however, several challenges need to be surmounted before these technologies can be used in applications, including the design of low-loss broadband structures with individual unit cell control. Ultimately, the realization of an optimal universal metasurface will rely on inverse design which is part of an overall hierarchical co-design of the active metasurface components: from the desired dielectric function of an active layer to an optimization of the unit cell shape as well as the configuration of the external stimuli. Multi-objective optimization algorithms could further support the design of metasurface unit cells that are robust to fabrication imperfections. To this end, an active area of research will consist of the development of computationally efficient algorithms. Supplementing the algorithms with physics-based models and constraints will enable efficient search and optimization of experimentally feasible design spaces [133].

**Table 2:** Summary of experimentally demonstrated values for the intensity modulation efficiency *η*, phase modulation, and the corresponding reflectance (*R*) or transmittance (*T*) at the largest phase shift, as well as the modulation frequencies and the operation wavelength regime. The modulation frequency refers to the highest frequency at which the amplitude response of an active metasurface resembles the applied signal. All reported values are for nanophotonic structures operating in reflection unless otherwise noted.

Modulation	Intensity	Phase modulation	Reflectance *R* or	Modulation	Operation
mechanism	modulation efficiency *η*	(with corresponding *η*)	transmittance *T*	frequencies	wavelength
			largest phase shift		
Field-effect based on	96% [37]	360° (*η* = 48%) [40]	8% Refl. [28]	30 MHz [127]	1510 nm [28]–
carrier modulation			4% Refl. [40]		THz [19]
Field-effect based on	50%^a^ [59]	42° (*η* = 29%)^a^ [65]	45% Refl.^a^ [65]	625 MHz^a^ [65]	620 nm [64]–
excitonic transition					755 nm [60]
Electro-optic tuning	37% Chromophores	70° (*η* = 73%) MQW [87]	5% MQW Refl. [87]	5 GHz [82]	915 nm [87]–
(inorganic metal-oxides,	(Transm.) [20]				1550 nm [76]
organic chromophores,	40% LN [76]				
multiple quantum wells)	73% MQW [87]				
Liquid crystals	80% (Transm.) [26]	180° Transm. (*η* = 75%) [128]	36% Transm. [108]	40 kHz [112]	460 nm [110]–
		360° Refl. (*η* = 38%) [110]			GHz (Transm.) [129]
Phase change and	82% GST [130]	180°(*η* = 78%) [25]	20% Refl. [25]	20 THz [115]	0.38 μm [131]–
phase transition	88% GSST [115]				THz (Transm.) [132]
materials	78% VO_2_ [25]				

^a^Reported value was obtained using either a heterostructure or a spatial light modulator.

### 2.2 Control architecture

To shape arbitrary wavefronts in space, a universal active metasurface needs to be fully reconfigurable in two dimensions. This is of particular interest for many imaging and communication applications, which may require directional scanning of beams across a scene. Two-dimensional control further enhances the information processing capability of an optical computing metasurface by dramatically increasing the number of independently addressable unit cells. However, this increased number demands sophisticated control architectures that allow for compact chip packaging with subwavelength unit cell spacings. Additionally, the control architecture should have minimal interference with the optical response of an active metasurface, *i.e.*, ideally the control system should not cause any degradation in the dynamic range of tuning or the attainable efficiency. Most demonstrations of active metasurfaces to date have consisted of a collective modulation of an entire array of scatterers [73, 93, 114] or addressing of individual unit cells along one dimension of the array (while connecting the scatterers along the perpendicular direction) [28, 40]. In the following, we highlight the active modulation schemes most suitable for achieving two-dimensional control based on the photonic modes and the nature of the external stimulus. We then discuss several pathways of designing an appropriate control architecture and evaluate the impact the respective approaches may have on the performance of an active metasurface.

To achieve an active metasurface that can be controlled in two dimensions, the resonant photonic mode needs to be confined along the lateral, longitudinal, and vertical dimension of the metasurface. This has been achieved in various active metasurface platforms using geometrically resonant scatterers in field-effect tunable metasurfaces [28] or liquid crystal-based structures [108]. In the case of a guided mode resonance [87], an extended mode is formed. Thus, independent control of metasurface elements can only be performed along the direction perpendicular to the guided mode, limiting this approach to one-dimensional spatial modulation. Similarly, active non-local metasurfaces [93] rely on phase modulation over length scales that are larger than the wavelength (and thus an individual metasurface element), but potentially smaller than the metasurface aperture. While this does not necessarily exclude two-dimensional control, additional research efforts are required to evaluate how non-local metasurfaces can be used for arbitrary wavefront shaping.

Additional constraints toward the realization of a two-dimensional control architecture are imposed by the nature of the external stimulus, which can be electrical, thermal, optical, chemical, or mechanical. Mechanical deformations, that are caused by, for example, stretching of an elastic substrate [134, 135], have previously been used to demonstrate two-dimensional beam focusing. However, prior research relied on changes that are introduced over the entire array configuration. As a result, the realized devices are restricted in terms of achieving different functions using a single chip. Similarly, it is challenging to confine changes induced using external chemical sources (such as the hydrogen or oxygen flow in hydrogenation metasurfaces [23]) or thermal sources [26, 136] to subwavelength spaces. Advanced schemes to inhibit the interference of neighboring unit cells would be necessary, requiring extensive multiphysics analysis and design. In comparison to these concepts, all-optical modulation relies on a pump-probe experiment, in which an intense pump pulse generates free carriers in the active medium and thus alters the properties of the scattered probe pulse [137]. Alternatively, interference of the two beams [138] or optical pumping of metasurface elements [139] can introduce structural and refractive index changes in the active medium. In either case, the resolution of the spatial pattern generated on the metasurface is given by the diffraction-limited spot of the pump laser. The requirement of additional pump lasers and beam scanners to write individual elements, however, inhibits a compact integration.

Over recent years, electrical biasing of metasurface elements has emerged as a promising pathway to enable two-dimensional control on a single unit cell level. The introduction of localized effects using an electrical stimulus has led to the development of several technologies ranging from electrically induced structural changes in phase change materials [130, 140] or liquid crystals [108] to mechanical deformations of scattering elements [16]. Due to its versatility in being able to be integrated with several modulation schemes, the following discussion aims to highlight potential pathways toward realizing a two-dimensional electrical biasing network and their respective challenges.

**Figure 4:**
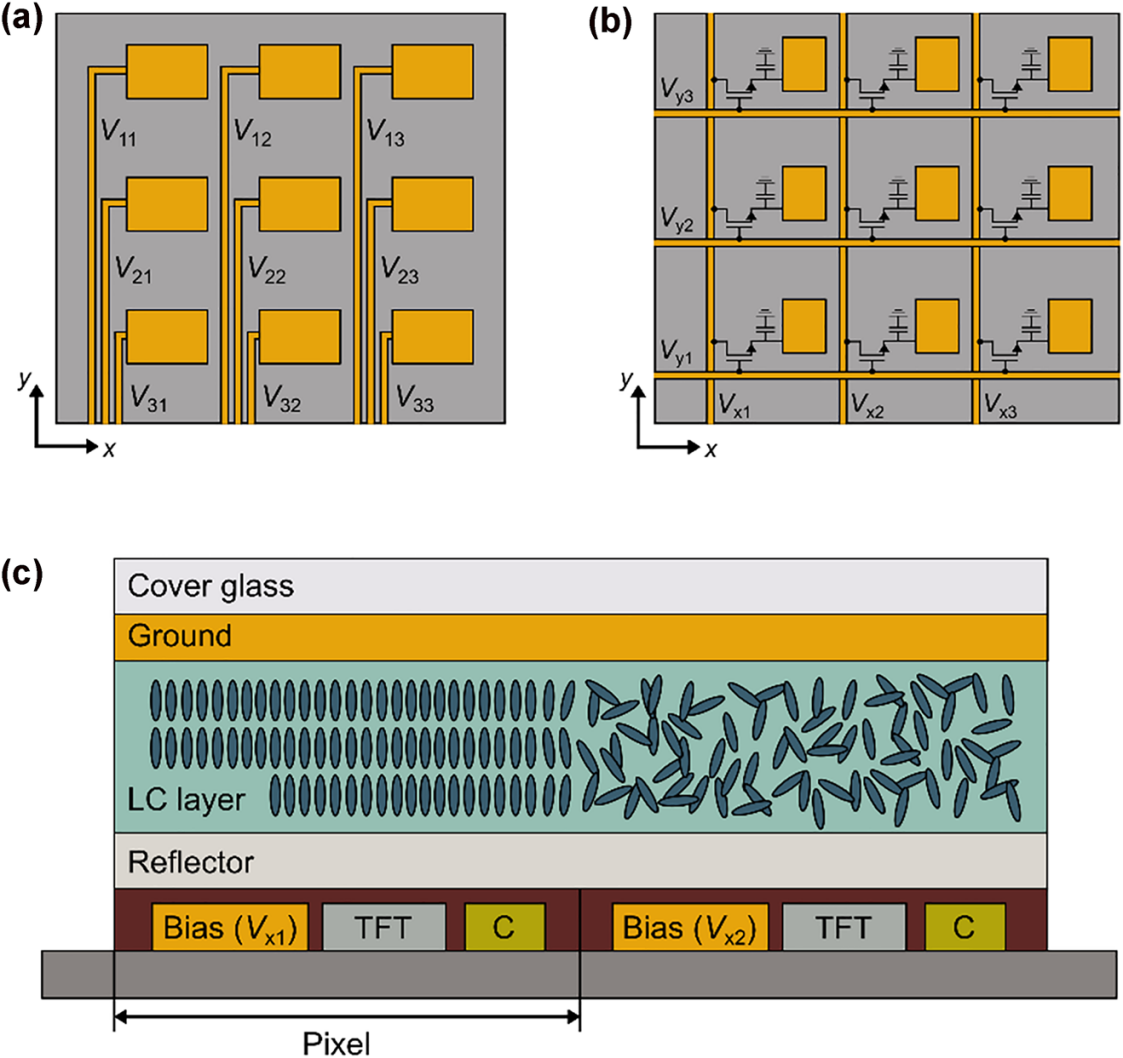
(a) Biasing of individual elements with voltage *V*_mn_ in a *m* × *n* array, where *m* and *n* are the number of rows and columns, respectively. (b) Row-column tuning of elements using biasing lines *V*_xn_ and *V*_ym_. A switching transistor and storage capacitor are built in at each element. (c) Cross-section across two pixels of a liquid crystal-based spatial light modulator relying on DRAM. The backplane consists of the biasing line, a thin film transistor (TFT) and a capacitor (C). These elements are covered by a reflector, the liquid crystal (LC) layer, the ground electrode, and the cover glass.

One route to designing an interconnect architecture for a two-dimensional active metasurface is to create individual biasing lines for each metasurface element. Kim et al. [46] recently experimentally demonstrated two-dimensional wavefront control using a plasmonic, field-effect tunable metasurface. In this work, fan-outs were used to electrically address each individual unit cell (Figure 4a). The gate electrodes were designed orthogonal to the scatterer orientation, as illustrated in Figure 4a. This configuration results in minimal perturbation of the resonance when the scatterers are excited with linearly polarized light in the *y*-direction. A common challenge with a biasing architecture like this, however, is its scalability. For metasurfaces with thousands or even millions of scattering elements, larger spacings between unit cells are required due to routing complexities. To overcome this limitation without sacrificing the metasurface aperture, multiple scatterers can be connected to one electrode, as shown in [46]. Notably, as the unit cell size approaches the incident wavelength, the field-of-view of the metasurface is strongly reduced. Additionally, an increased amount of undesired scattering is expected for coarsely resolved phase profiles, as previously shown for gradient beam steering metasurfaces [28].

An alternative strategy relies on row-column or perimeter tuning of individual unit cells. This approach involves connecting the metasurface elements in individual rows and columns (Figure 4b), respectively, allowing for a dramatic reduction of biasing lines from *N* ^2^ (for individual unit cell control) to 2*N* for an array consisting of *N* × *N* unit cells. Row-column tuning is commonly used in commercial spatial light modulators consisting of large arrays of pixels (>1000 × 1000 elements). Spatial light modulators comprise non-resonant pixels relying on a phase accumulation in thick liquid crystal layers [141]. Arbitrary wavefront shaping is achieved using either a dynamic random-access memory (DRAM) or a static random-access memory (SRAM) circuit [141, 142]. The backplane of a DRAM unit cell consists of the biasing line, a single thin film transistor (TFT) and a capacitor to switch and store the state of an element, respectively. Figure 4c illustrates a schematic cross section across two pixels of a liquid crystal-based spatial light modulator in a DRAM architecture. By periodically scanning the biasing line in each row and column, respectively, the capacitor at each pixel is charged with the required voltage. SRAM circuits, in comparison, are purely digital and built using multiple logic gates per element to encode the desired voltage. Consequently, DRAM architectures allow for smaller pixel sizes that are usually in the range of 2–10 μm [142, 143].

To achieve high resolution wavefront control with a wide field-of-view, researchers are pursuing strategies to further downscale the pixel size in spatial light modulators. Conventionally, the biasing line, the switching thin film transistor (TFT), and the storage capacitor are arranged laterally in one plane, as schematically shown in Figure 4c. The TFT channel length cannot be further reduced in this configuration, as a concentration of large currents into small regions causes an increase in temperature that leads to channel degradation [111]. To overcome this limitation, vertically stacked thin film transistors (VSTs) and vertical channel thin film transistors (VTFTs) have been proposed [111, 144], [145], [146]. A VST architecture relies on an overlap of the biasing line and the TFT/capacitor elements to reduce the pixel area. Using this approach, Choi et al. [144] realized spatial light modulators with pixels as small as 1 μm in the lateral direction. VTFTs allow additional downscaling of the pixel in the longitudinal direction by forming the channel along the sidewall of the vertical structure [111, 145, 146]. The main challenge of using this approach is the patterning and material deposition required to obtain the vertical structure [111].

In comparison to spatial light modulators, metasurfaces consist of resonant scattering elements. As a result, the phase accumulation occurs due to the scatterer rather than the liquid crystal layer thickness. Liquid crystal-based metasurfaces therefore support reduced cell thicknesses that allow operation under smaller applied voltages. The smaller liquid crystal layer thickness additionally allows for shorter response times. Li et al. [147] recently realized a two-dimensional liquid crystal-based metasurface based on row-column tuning with a pixel size of 1 × 1 μm^2^. It is to be noted that the reduced liquid crystal layer thickness in metasurfaces limits the effects of fringing electric fields, which degrade device performance due to smeared out phase profiles [148, 149]. Nevertheless, the elastic motion of the molecules, which leads to crosstalk between adjacent unit cells, limits further reductions in pixel size [111]. In comparison, active materials that are used in field-effect or electro-optically tunable metasurfaces do not face this limitation (with the appropriate design of a localized resonant mode). The advancements in downscaling pixels using vertically stacked or vertically oriented TFTs could provide a potential pathway for the integration of subwavelength DRAM circuits into active metasurfaces. Rather than having a continuous ground plane, the back reflector of an active metasurface would need to be transformed into a ground gate electrode oriented perpendicular to the top electrode (Figure 4b). In addition, a transistor and capacitor need to be integrated to enable sequential scanning of rows/columns. Non-volatile phase change memory materials [150, 151] could provide a promising platform to introduce the required latency into the interconnect architecture without the need of an additional capacitor. However, elaborate thermal analyses need to be performed to ensure that there is no thermal crosstalk between adjacent elements.

A crucial aspect in the design of electrical circuits for row-column tuning is the dwell time required to configure or change the state of each metasurface element. The added dwell time is expected to have a direct impact on the accessible modulation frequencies of active metasurfaces. Fast scanning of biasing lines would, in principle, enable applications such as holographic displays, where our eyes receive an image that is averaged over fractions of a second. However, the additional time required to scan all row/column elements across large two-dimensional arrays may potentially limit access to the time-modulated operation regime of an active metasurface. This becomes particularly relevant for modulation mechanisms that support frequencies beyond the MHz regime. An alternative approach suggested row-column tuning without a capacitive element [152]. This would, in principle, allow driving frequencies of several MHz to GHz based on the modulation mechanism. In this design, however, the attainable functions of an active metasurface are restricted to those that can be represented as a convolution of two linearly independent responses associated with the orthogonal directions of the array. As a consequence, this approach inhibits arbitrary wavefront shaping.

In summary, while there are existing interconnect architectures for biasing of individual unit cells, the designs implemented in active metasurfaces to date are not scalable. Row-column tuning using a capacitive element offers an alternative approach with a drastic reduction in biasing lines. Existing TFT technologies for spatial light modulators have been downscaled to a pixel size of ∼1 μm^2^. For operation in the visible, however, further reductions in size are required to increase the field-of-view. Thus, careful redesign of the biasing network and analysis of interelement crosstalk is required to develop an appropriate interconnect architecture for two-dimensional control. Additional studies are needed to understand the impact of dwell time reconfiguration on time-modulated operation. Table 3 provides a summary of challenges toward the realization of a two-dimensional active metasurface based on row-column tuning (with latency). Further, we would like to note that most reported works on active metasurfaces and spatial light modulators in this section have relied on operation in reflection only. Therefore, we need to explore alternative materials and biasing architectures that have a minimal impact on the output efficiency and thus enable metasurface operation both in reflection and in transmission. An important consideration for the realization of two-dimensional active metasurfaces is its CMOS-compatibility, which would be required to utilize existing fabrication techniques in commercial semiconductor foundries. Finally, we note that as we move to large-scale active metasurfaces, we need to rely on low-cost, high-throughput fabrication techniques, such as immersion lithography [153], nanoimprint lithography [154], or rolling mask lithography [155].

**Table 3:** Challenges toward the realization of a row-column tuning approach for various modulation mechanisms that rely on an electrical stimulus.

Modulation mechanism	Challenges toward row-column tuning (w\latency)
Liquid crystals	Crosstalk in subwavelength pixels due to elastic motion of molecules
Field-effect tuning	Potential reduction in modulation frequency based on dwell time
Electro-optic tuning	Potential reduction in modulation frequency based on dwell time
Electro-mechanical deformations (MEMS/NEMS)	Interference of mechanical motion with biasing lines Potential reduction in modulation frequency based on dwell time
Phase change materials	Thermal crosstalk in subwavelength pixels Potential reduction in modulation frequency based on dwell time
Phase transition materials	Thermal crosstalk in subwavelength pixels

### 2.3 Advanced operation modes

Successfully realizing a universal active metasurface would have significant impacts upon many research directions currently being pursued within the field of optics. In the following, we discuss the limitations of existing technologies in the fields of optical imaging, communication, and computation, and explore the novel applications that could be realized with the added capabilities of a universal active metasurface.

#### 2.3.1 Optical imaging

Optical imaging is a crucial component of realizing many next-generation technologies, including autonomous vehicles, machine vision for drones, biomedical and diagnostic techniques, holography, and quantum information technologies. One of the most important building blocks of optical techniques is the lens. Whilst conventional lenses have been able to demonstrate desirable optical imaging techniques, they are bulky, heavy, and expensive, preventing them from being integrated into wearable and lightweight devices. Metalenses, on the other hand, exhibit a significantly reduced form factor, allowing for their integration into these devices. While the current cost of fabrication for metalenses is considerably higher than their bulky counterparts, metalenses have the potential to exhibit exotic functionalities, such as achromatic or 3D imaging, through precise engineering of their subwavelength elements.

Unlike conventional lenses, the resonant scatterers of passive metalenses are generally highly dispersive in nature. This leads to strong chromatic aberration away from a particular wavelength, and hence they have usually been designed for single-wavelength operation [156–158]. Recently, several metalenses capable of focusing a set of discrete wavelengths without chromatic aberration [156, 159] and over a finite continuous wavelength range [7, 160], [161], [162], [163], [164], [165] have been demonstrated. One approach that has been adopted to realize this extended achromaticity is to use integrated-resonant unit elements (IRUEs), in which coupled metallic nano-rods supporting multiple plasmonic resonances provide a linear phase profile with 1/*λ* [164]. However, the diameters of these passive metalenses are limited to the order of 100 μm by the requirement for large group delays [163], limiting their spatial resolution and performance. Furthermore, as these are passive systems, the focal lengths are fixed and cannot be varied post-fabrication. Varifocal metalenses using active metasurfaces have been demonstrated through field-effect tuning [28], integrating phase-change materials [166] and mechanical modulation with elastic substrates [167]. However, these metalenses are typically single-wavelength operation, as they suffer from chromatic aberrations. This is due to a non-linear relationship between the phase of the scatterers and wavelength, stemming from insufficient control over their phase profiles. Thus, a universal metasurface with arbitrary phase control of its elements could potentially exhibit dynamic focal-length tuning whilst potentially replicating the appropriate phase profiles of the passive metasurface elements, leading to the realization of a tunable achromatic metalens.

One optical technique that has been realized with passive metalenses is three-dimensional (3D) imaging. To date, depth-sensing has been realized using two main approaches: light-field imaging [168, 169] typically uses multiple metalenses, either interleaved or off-set from each other. Alternatively, engineering of point spread functions (PSFs) [170–173] relies on metasurfaces with complimentary PSFs to achieve both axial and lateral sensitivity. However, these approaches tend to suffer from a combination of poor spatial resolution, poor axial resolution, limited field of view, limited depth of focus and/or limited wavelength range, depending upon the device. Importing the universal metalens discussed in the previous paragraph into 3D optical imaging systems would offer several advantages over current devices. First, reconfigurable metalenses could be programmed to perform functions sequentially in time that are currently performed by spatially separated distinct components. Examples of this are taking several snapshots in different imaging planes or redefining the PSF. Thus, the entire aperture of the device could be utilized for each function, resulting in improved spatial lateral resolution. Furthermore, the continuous tuning of the focal length would offer higher axial resolution than passive devices with a set of discrete focal lengths. A universal active metasurface could also allow some degree of tuning over the depth-of-focus and wavelength operations ranges, so that a single device could be used for multiple applications.

Another sought-after technique in optical imaging is holography. Passive and active metasurfaces have been used to demonstrate static [174–176] and dynamic [23, 177], [178], [179] holography. However, many of the previous demonstrations of dynamic holography involve switching part of the hologram on or off, often through chemical reactions. To the best of our knowledge, there has yet to be a demonstration of a truly reconfigurable hologram metasurface at optical wavelengths, in which any arbitrary two-dimensional image could be displayed. To achieve this feat, precise dynamic control over each individual unit cell would be required. Moreover, a metasurface capable of displaying three-dimensional holographic images would need to exhibit control over both amplitude and phase independently [180]. Thus, the universal metasurface would be an ideal platform for demonstrating two- and three-dimensional holograms that could be dynamically and arbitrarily changed. Furthermore, the universal metasurface could potentially remove anomalous speckles from images and demonstrate sophisticated holographic techniques such as rendering surface textures of 3D holographic objects through its independent control of phase and amplitude, realizing a quality of holographic display not achievable with current technology.

#### 2.3.2 Optical communication

Space-time or time-modulated metasurfaces are a class of active metasurfaces that simultaneously impart a spatial and temporal phase gradient to incoming light [181–183]. Their control over the spectral content of the scattered light has made them an exciting candidate for many applications in optical communications from LiDAR to deep space communications. While the concept of time-modulated metasurfaces has previously been demonstrated in the radiofrequency (RF) domain, it has only recently become feasible in optical frequencies with the realization of active metasurfaces with decreased response times, allowing for higher modulation frequencies [30, 181, 184, 185]. Recently, there has been a surge of interest in the photonics community to realize time-modulated metasurfaces in the optical domain because of their ability to: 1) create a dispersionless spatial phase gradient with constant amplitude, 2) enable multi-channel communication in a single aperture, and 3) exhibit nonreciprocal behavior.

As discussed in the *Metasurface design* section of this Perspective, many active metasurfaces suffer from the coupling of phase and amplitude which limits the efficiency of a given optical function and creates unwanted scattering of light, especially if 2*π* phase span is not achieved in the quasi-static domain. In space-time metasurfaces, the conversion efficiency from the fundamental frequency to the harmonic of interest is still limited by amplitude-phase coupling [182]. However, the spatial phase can be tuned across a 2*π* range by shifting the phase of the applied waveform at each metasurface element using a series of external phase shifters. This allows for the creation of a nonresonant, dispersionless phase shift with constant amplitude, which can effectively overcome the challenges associated with coupled phase and amplitude in quasi-static active metasurfaces.

**Figure 5:**
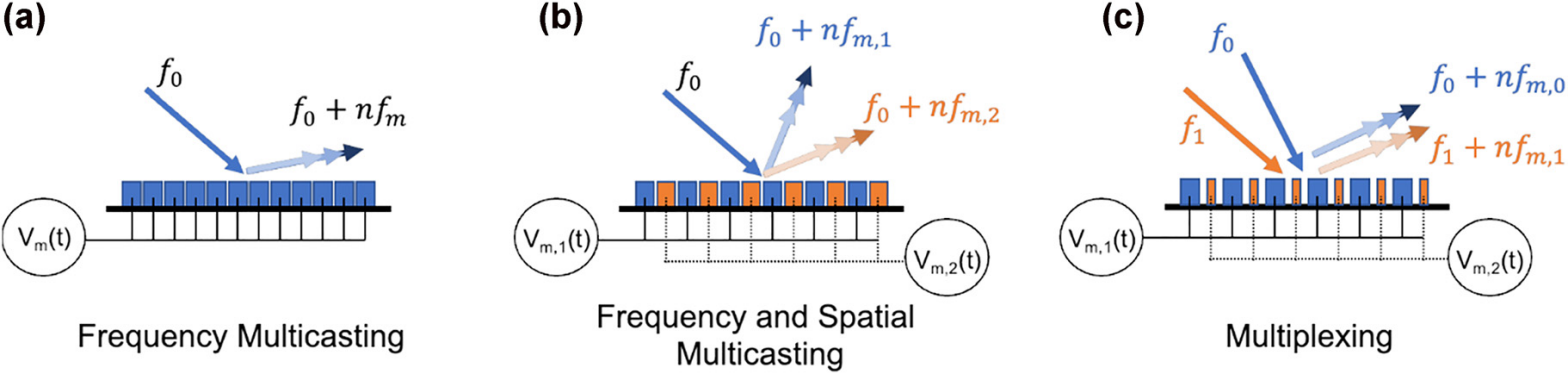
Multicasting and multiplexing schemes achievable with a space-time metasurface illuminated with optical frequencies and modulated with RF signals. In all schematics, *V*_*m*_(*t*) denotes a time-varying voltage of frequency *f*_*m*_ applied to the metasurface, *f*_0_ is the frequency of light incident on the metasurface, and the output light is a sum of the incident frequency with integer multiples of the modulating frequency. (a) A single input is converted to multiple frequencies and steered to a given angle. (b) A single input frequency incident on a space-time metasurface driven with two waveforms of differing frequency and spatial phase gradient. This results in two spatially tunable beams, each consisting of a different spectrum of harmonics. (c) A space-time metasurface consisting of two alternating elements with different resonant frequencies. This allows two input frequencies to be individually steered, while controlling the harmonic content of each beam.

Another capability that is realized by space-time metasurfaces is multicasting and multiplexing of information [181, 182, 185, 186]. Multicasting refers to sending a single input to multiple outputs, separated either in space or frequency. Multiplexing encodes multiple inputs into a single output wave that can be demultiplexed back into the original signals at the receiver. Figure 5 illustrates possible multicasting and multiplexing schemes achievable with a time-modulated metasurface operating at optical frequencies and modulated with RF signals. It is important to note that, in general, the frequency harmonics generated by a space-time metasurface will be steered to different angles, regardless of the spatial phase-gradient. However, because the attainable modulation frequencies in state-of-the-art active metasurfaces are multiple orders of magnitude smaller than optical frequencies, the angular separation between generated harmonics will be negligible and for the purpose of this discussion, we will ignore this angular separation. Figure 5a shows a simple frequency multicasting device that accepts one input frequency, generates new harmonic sidebands, and uses a subwavelength spatial phase-gradient to direct the output light to a given angle. For such a device, all metasurface elements are identical and modulated with the same RF waveform, phase-shifted in space. Extending this concept, one can apply two alternating waveforms to every other metasurface element to achieve a more complex multicasting functionality, depicted in Figure 5b. Here, the temporal and spatial phase-gradients of each modulating waveform can be altered to tune the angle and spectral content of the two scattered wavefronts. Finally, by using metasurface elements with different resonance wavelengths [187], multiple input frequencies can be accepted and multiplexed to multiple independently tunable output channels, as depicted in Figure 5c. While the spatial phase gradients depicted in Figure 5 are assumed to be subwavelength blazed gratings, alternative optical functions can be realized for each wavefront by designing appropriate spatial phase configurations. The ability to control multiple channels simultaneously increases the amount of information that can be sent and received with optical frequencies in a single aperture.

Lastly, another exciting capability of space-time metasurfaces is their ability to violate Lorentz reciprocity by breaking time-reversal symmetry [30, 181, 183, 188]. The spatio-temporal modulation of refractive index creates an effective motion of phase within the device, causing incoming light to see a direction-dependent phase gradient. This effect is commonly demonstrated by applying a large in-plane magnetic field, requiring a bulky ferromagnetic magnet. Space-time metasurfaces can break time-reversal symmetry in a compact and lightweight device, increasing the range of possible applications. A simple device that is enabled by this functionality is a unidirectional beam splitter which acts as a conventional beam splitter when illuminated from one side and allows light to pass normally when illuminated from the other. Additionally, the nonreciprocal separation of angles between incident and transmitted signals can allow for full-duplex communication: a communication network consisting of devices that can simultaneously send and receive information. Interference between received and transmitted signals is a common problem that limits full-duplex communication. This is solved by designating one frequency to receive signals and another to transmit, increasing the complexity and cost of the overall device.

Space-time metasurfaces allow for full 2*π* coverage with constant amplitude, multicasting and multiplexing functionalities, and nonreciprocal behavior, enabling various complex communication functionalities in a single aperture. Such compact devices are highly desirable in applications such as LiFi, LiDAR, and space communication.

#### 2.3.3 Optical computation

Due to their control over phase and amplitude in space and time, a great amount of research has been dedicated to metasurfaces for optical computing. The field of optical computing has been an active research topic for many decades but the reliance on nonlinear phenomena to create logic gates (requiring high power densities and long propagation distances) has prevented optical processors from surpassing the performance of their electronic counterparts [189]. Despite its limited use in industrial applications, optical computing has many inherent advantages over electrical computing such as capability for highly parallelized processing, high rate of data transfer, and low power requirements [190, 191].

Recently, metasurfaces have been used as tools for optical signal processing to perform functions such as edge detection, differentiation, and solving differential equations [192–196]. In these devices, one of two techniques is commonly employed to processes input information: the use of spatial Fourier transforms or Green’s function [190]. In the spatial Fourier transform approach, a 4*f* lens system is used with a precisely designed metasurface spatial filter at the Fourier plane of the two lenses. In contrast, demonstrations using Green’s function devices directly encode the desired transform to a single metasurface layer, without using lenses to take the Fourier and inverse Fourier transforms. In their pioneering demonstration, Silva et al. [193] implement both techniques to take spatial derivatives of an input waveform. Their first device uses a metasurface sandwiched between two gradient index (GRIN) lenses. The GRIN lenses take Fourier and inverse Fourier transforms and the metasurface is specially designed to take the first derivative of the input signal. In contrast, the authors also used a multilayered metamaterial slab to create the Green’s function associated with a second derivative. By directly implementing this function, the device does not need to work in the Fourier space and removes the requirement of the GRIN lenses. Many variations of these techniques have been demonstrated recently, further illustrating the computational capabilities of metasurfaces [197–200].

In all works discussed thus far, a passive metasurface or metamaterial is designed for a single functionality. A universal tunable metasurface would provide the freedom to control the transfer function of a device even after fabrication. A single metasurface could be used as an effective ‘optical calculator’ to, for example, impart a spatially dependent multiplicative factor on an input waveform, then be reconfigured to take the derivative of a different waveform. This freedom greatly increases the applications of a single metasurface. In addition, arbitrary control over all properties of light allows for a general transfer function in a single metasurface layer. A recent study by Kulce et al. [191] investigated the fundamental information limits of a single layer metasurface consisting of individually controllable complex valued transmission coefficients. This work considered a signal from an input aperture, sent through a series of linear transforms, and received at an output aperture. The authors showed this system could be reproduced by a single universal metasurface consisting of at least *N*_*i*_ × *N*_*o*_ individually addressable elements, where *N*_*i*_ and *N*_*o*_ are the pixels of the input and output apertures, respectively. Thus, for a universal active metasurface with a sufficiently large aperture and density of individually addressable elements, any series of linear transfer functions can be implemented and changed in time, in a single layer. While implementing a finite number of linear transforms in series is useful, many scientific problems require a feedback loop and iterate toward a solution. For example, solving differential equations without an analytic solution and machine learning algorithms typically start with an initial guess, adapt this guess, and repeat until a solution is obtained which meets certain accuracy criteria. To implement such algorithms optically, we envision integrating a universal metasurface in a cavity and adapting its amplitude/phase profile over time until a solution is achieved. An alternative to this approach could be to cascade a finite number of metasurfaces in space. While this arrangement has a limit on the number of times an input signal interacts with a metasurface layer, interesting functionality could be explored by designing the device to create near-field coupling between the metasurface layers. Such a highly connected system could potentially be used to reproduce neural networks where each metasurface element represents a neuron and connections between neurons could be controlled via manipulation of the near-field coupling. These proposed devices represent forward-looking perspective of possible uses for a universal metasurface and illustrate the wide range of applications for metasurfaces in digital and analog optical computation.

## 3 Discussion & conclusion

Over the last few years, there has been remarkable progress toward dynamic manipulation of light using active metasurfaces. In this article, we highlighted several technologies that are particularly promising for the realization of a universal active metasurface for arbitrary, on-demand wavefront shaping. Table 2 provides a summary of the performance of state-of-the-art active metasurfaces in terms of their dynamic range of tuning, the attainable output efficiencies, the modulation frequency, and the wavelength coverage. While none of the current designs meets all target performance characteristics, ITO-based field-effect tunable multifunctional metasurfaces [28, 40, 46] currently most closely resemble the conceptual universal active metasurface. These metasurfaces have been used to demonstrate a diverse set of functions based on individual unit cell control. High modulation frequencies of field-effect tunable metasurfaces (in the range of several MHz [18]) further give access to advanced operation modes, which rely on the concept of space-time modulated metasurfaces. However, the large absorption observed in these gate-tunable plasmonic metasurfaces is a major drawback of this approach.

Active metasurfaces, which use electrically controlled reorientation of liquid crystal molecules, are also a promising pathway toward the realization of the universal metasurface. Liquid crystals offer an advantage of active tuning with low losses and are thus widely used in applications. Researchers have realized both reflective [110] and transmissive [26, 108] liquid crystal-based structures exhibiting a large dynamic range of tuning. Furthermore, multi-resonant designs have been proposed for operation across the visible spectrum [110]. A significant limitation, however, arises due to the slow response times of liquid crystal molecules, restricting the operation of such metasurfaces to the quasi-static regime. Electro-optic materials could therefore be an effective alternative for active metasurfaces supporting high modulation frequencies along with low losses. Current leading designs, however, are based on high quality extended modes [87] which, in principle, inhibit the perspective of two-dimensional control of the wavefront shape. Thus, novel designs of subwavelength high-*Q* metasurface elements need to be developed, which will localize the mode in three dimensions.

Another challenge which needs to be surmounted for the realization of a universal active metasurface is the design of an appropriate two-dimensional control network. Electrical biasing of metasurface unit cells has emerged as a promising pathway for individual unit cell control in a variety of modulation schemes. Here, the main challenge lies in developing electrical circuits that allow for subwavelength unit cell control in large arrays with thousands to millions of elements. The design of individual biasing lines for each metasurface element [46] provides an intuitive approach to realizing two-dimensional control in the near future. However, it is challenging to integrate this control architecture into compact subwavelength spacings. Thus, row-column tuning may provide an effective alternative to dramatically reduce the number of biasing lines. The latency required to scan and configure each element demands an integration of additional transistors and capacitors in the biasing network. In this aspect, continuous efforts made toward miniaturizing existing thin film transistor technology used in spatial light modulators [111, 144] may have a direct impact on the mechanisms in which next-generation active metasurfaces are configured.

Based on current challenges toward the realization of a universal metasurface, we anticipate the following areas to play a critical role in the development of versatile active metasurfaces:

1. *Advanced fabrication techniques and new materials:* Low-loss active materials supporting high modulation frequencies form a crucial component of a high performance universal metasurface. The current leading material platform in this aspect constitutes of electro-optic materials. Commercially available lithium niobate thin films have allowed for considerable progress toward achieving a large dynamic range of tuning. However, the corresponding metasurface designs rely on extended or non-local high-quality resonant modes to capitalize on the modest electro-optic refractive index changes. Further progress in the fabrication of thin crystalline films of electro-optic materials may enable their integration with alternative unit cell designs for individual element control. Alternatively, the search for new low loss materials and material compounds for integration into field-effect tunable metasurfaces may provide an effective pathway to obtaining power efficient active metasurfaces that support high modulation frequencies. In this context, machine learning-assisted materials discovery will play a critical role to find materials or material compounds that exhibit optimal complex dielectric functions for low loss operation [201–203].
2. *New metasurface design concepts:* On a unit cell level, a universal metasurface requires individual unit cell control, independent manipulation of all characteristic properties of light, and a tunable operation wavelength. Metasurfaces based on electro-optic materials require novel design strategies for developing subwavelength metasurface unit cells which can both exhibit high quality factors and localize the optical mode in three dimensions. Notably, resonant operation of active metasurfaces results in a coupled response of multiple properties of the scattered electric field, such as its amplitude and phase. This creates undesired effects in the wavefront of scattered light. Dual-gated metasurfaces, enabling the application of two independent voltages to a single unit cell, have therefore been proposed to demonstrate independent control over the amplitude and phase of the reflected light [38–40]. An extension of this concept to multi-gated structures could provide independent control over more than two characteristic properties of the scattered light. Alternatively, independent control over the scattered light properties may be realized by stacking metasurfaces, where each surface controls a different property of light [204]. Further advances in the design of efficient transmissive active metasurfaces would be instrumental for the realization of compact optical components consisting of metasurface stacks. Finally, further advances in the design of multi-spectral active metasurfaces are needed to obtain a tunable operation wavelength. This may involve designs of geometrically multi-resonant structures [110, 187], or an integration of different active materials into one unit cell.
3. *Multiphysics modelling for two-dimensional electrical control architectures:* Two-dimensional control of active metasurfaces is necessary to move active metasurfaces into the realm of practical applications and harness their full potential. In this regard, further efforts toward multiphysics modelling of the metasurface together with its electrical control network are required to evaluate the feasibility of different designs. In the case of a two-dimensional biasing network, the gate electrodes pass through the area where light is directly interacting with the resonant scatterer. This is different from the case of one-dimensional control, where metasurface unit cells are electrically biased from the side. Therefore, it is critical to analyze the impact of the control circuit on the optical response of a resonant metasurface element. This includes analyzing the impact of biasing lines on the overall dynamic range of tuning and device efficiency as well as potential crosstalk between high-frequency signals across closely spaced biasing lines. Additional studies addressing the electrical response times are required to determine inherent limits to the modulation frequencies arising from the employed interconnect architectures. Finally, a thermal analysis is needed to evaluate heat damage thresholds upon downscaling electrical components as well as thermal crosstalk effects between adjacent unit cells.
4. *Miniaturization of electrical control components and integration into metasurfaces:* In order to obtain high-resolution beam shaping with a large field-of-view, we need to develop large scale metasurfaces with subwavelength unit cells. Row-column tuning of a two-dimensional array provides an effective pathway to limit the number of biasing lines. However, the row-/column-wise reconfiguration requires a latency that is achieved through the integration of additional transistors and capacitors in the circuit. Further research is required to realize sub-micron scale electrical circuits that allow to switch and store the state of an individual element. Current metasurface designs then need to be adapted to incorporate the biasing network into each individual unit cell. A grand challenge in this aspect also lies in the realization of two-dimensional transmissive metasurfaces, which experience minimal loss in efficiency due to the biasing network.

With the realization of a universal active metasurface, we expect an explosion of novel applications in the realms of optical imaging, communication, and computation, some of which have been discussed in this article. An integration of the active metasurface with nanophotonic light sources, such as vertical cavity surface emitting lasers (VCSELs) [205, 206], may further lead to the emergence of chip-scale light sources that allow for arbitrary beam shaping in real-time. Moreover, coupling of the universal metasurface to quantum emitters will enable the realization of novel quantum devices that could be used to capture and manipulate the state of single photons [207, 208]. For efficient use in applications, we ultimately foresee using deep learning approaches to identify the optimal control sequences applied to the metasurface, resulting in the desired optical response. The ability to encode active metasurfaces using advanced computational methods will thus enable the creation of a universal optical processing unit that can function independently and reprogram itself based on the desired task at hand.
